# Sandwich-Structured Silver Nanowire Transparent Conductive Films with 3H Hardness and Robust Flexibility for Potential Applications in Curved Touch Screens

**DOI:** 10.3390/nano9040557

**Published:** 2019-04-05

**Authors:** Xikun Chu, Jingqi Tao, Shuxin Li, Shulin Ji, Changhui Ye

**Affiliations:** 1 Key Laboratory of Materials Physics, Anhui Key Laboratory of Nanomaterials and Nanotechnology, Institute of Solid State Physics, Chinese Academy of Sciences, Hefei 230031, China; xikunchu@163.com (X.C.); jqtao127@163.com (J.T.); lishuxin@issp.ac.cn (S.L.); 2 Science Island Branch of Graduate School, University of Science and Technology of China, Hefei 230026, China; 3 College of Materials Science and Engineering, Zhejiang University of Technology, Hangzhou 310014, China

**Keywords:** silver nanowire, sandwich structure, hardness, adhesion, haze

## Abstract

A sandwich-structured bottom hard-coat/silver nanowire/top hard-coat (BHC/AgNW/THC) transparent conductive film (TCF) has been prepared by embedding the functional AgNW layer between two HC layers. The BHC/AgNW/THC TCFs show high scratch resistance with a hardness of 3H due to the enhanced adhesion to the substrate. In addition, the BHC/AgNW/THC TCFs exhibit a transmittance of 90.6% and a haze of 1% at 550 nm under a sheet resistance of 72 Ω/sq. Furthermore, highly enhanced long-term stability has been guaranteed by the HC layers due to their excellent gas barrier property. The amazing fact is that hard coating has little effect on the flexibility of AgNW films especially under extreme bending conditions and negligible resistance change could be observed after bending over thousands of times. Consequently, the greatly improved performance of BHC/AgNW/THC TCFs provided by employing hard coating layers paves the way for real-world applications of flexible AgNWs in vast areas that rigid indium tin oxide is not suitable.

## 1. Introduction

Transparent conductive films (TCFs) are essential for many optoelectronic applications including photovoltaic cells [1], organic light-emitting diodes [2], touch screens [3], and film heaters [4]. Indium tin oxide (ITO) in TCFs is the most prevalent material used for electronics [5]. However, the scarcity of indium results in the unstable cost of ITO materials. In addition, ITO is fragile and not suitable for flexible circuits. Therefore, several other candidates such as carbon nanotubes (CNTs) [6], graphene (GR) [3,7], conducting polymers [8], metal mesh [9], and metal nanowires [10,11] have been extensively researched as possible materials to substitute for ITO. One of these includes silver nanowire (AgNW) TCFs, which are among the materials with the best suitability for making flexible transparent electrodes because of their high optical transparency, conductivity and structural flexibility [12,13].

Hardness is essential for a TCF in practical applications [14]. However, the hardness of AgNW TCFs does not meet the requirement due to two reasons. Firstly, solution-processed AgNW networks are loosely deposited on a substrate due to the weak interaction between AgNWs and the substrate; secondly, AgNWs are soft and cannot resist a scratch with sharp tip. To overcome these problems, some methods have been proposed, by applying CNTs, GR or metal oxides as a protective layer to increase scratch resistance of AgNW TCFs [15,16,17]. Unfortunately, these protective layers on AgNWs reduced the transmittance to some extent. By encapsulating with a thin layer of polymer [18,19,20] or introducing covalent bonds [21] between AgNWs and the substrate, these methods showed an adhesion improvement, but limited hardness improvement due to the soft property of the introduced materials, such as poly-(3,4 ethylenedioxythiophene)/poly-(styrenesulfonate) (PEDOT:PSS) or poly-dimethyl-siloxane (PDMS). The improvement of hardness and scratch resistance in the above methods is limited, and it is still necessary to find solutions for making highly scratch-resistant AgNW TCFs for applications including touch screens, outdoor heaters, and human–computer interactive sensors. Therefore, it remains a challenge to develop a highly efficient approach to improve hardness and scratch resistance of the AgNW TCFs.

Herein, we present a sandwich-structured bottom hard-coat/silver nanowire/top hard-coat (BHC/AgNW/THC) TCF in which the AgNW layer is embedded between two hard coating layers. The BHC/AgNW/THC TCFs show high scratch resistance with the hardness of 3H, and enhanced adhesion to the substrate. In addition to this, high transmittance of 90.6% and low haze of 1% at 550 nm under low sheet resistance (72 ± 4.0 Ω/sq) as well as long-term stability in air have been exhibited. Negligible resistance change has been observed after bending over thousands of times.

## 2. Materials and Methods 

### 2.1. Materials

Silver nitrate (AgNO_3_, ≥99.8%) was purchased from Shanghai Qiangshun Chemical Reagent Co., Ltd. (Zhabei, SH, China). Ethylene glycol (EG), NaCl, NaBr, NH_3_·H_2_O and polyvinylpyrrolidone (PVP, molecular weight of 360,000) were purchased from Sinopharm Chemical Reagent Co., Ltd. (Shanghai, China). Acrylic resin (25 wt% RSH-P2015) as a BHC was obtained from Taiwan Poly Technology Co., Ltd. The main component is polymethyl-methacrylate in toluene and butanone (volume ratio of 4:1). Acrylic resin of 1.2 wt% concentration as a THC was prepared by diluting acrylic resin (25 wt% RSH-P2015) in isopropyl alcohol. The UV curing reaction was carried out in a UV curing chamber (IntelliRay 400, Shenzhen Wisbay M&E Co., Ltd., China) with a fully enclosed curing environment, all-points shielding by maximizing UV protection to minimize oxygen inhibition [22]. A four-sided applicator (FSA, SZQ5-20) was obtained from Hefei Kejing Materials Technology Co., Ltd. (Hefei, China). The FSA was a precise film scraper with four surfaces, and each surface had a coating thickness of 5, 10, 15 and 20 µm, respectively. Cleaned flexible polyethylene terephthalate (PET) films were used as substrates.

### 2.2. Synthesis of AgNW 

The synthetic method was based on the previous report by our group [23]. First, NaBr solution (0.0114 g in 0.5 mL of EG), NaCl solution (0.0123 g in 1 mL of EG), PVP-360000 solution (0.2800 g in 5 mL of EG) and fresh AgNO_3_ solution (0.2255 g in 5 mL of EG) were separately prepared. The solutions were combined and added to a 100 mL flask with 38.5 mL of ethylene glycol. The mixed solution was stirred appropriately for 30 min. Then the flask was heated to 180 °C within 10–20 min in an oil bath. The bubbling reaction was carried out with nitrogen during the heating process (gas flux of 150 mL min^−1^). When the temperature reached 180 °C, the solution was stationarily and naturally cooled to 160 °C. Thereafter, the reaction was maintained for 2 h, and then the solution was cooled to room temperature to obtain the solution of AgNWs. The synthesized solution was washed several times with a large amount of NH_3_·H_2_O to remove large nanoparticles (AgCl and AgBr) and with acetone to remove small silver nanoparticles. The purified AgNWs were obtained with an average diameter of 21 nm and an average length of 34 μm, respectively. Finally, the purified AgNWs were formulated into a stable ink (1 mg/mL).

### 2.3. Formation of BHC/AgNW/THC TCF

An FSA (coating thickness of 15 µm) was utilized to coat acrylic resin (25 wt% RSH-P2015) on PET substrates (100 µm). A BHC with a hardness of 3H was obtained by drying at 80 °C for 1 min and UV irradiating for 10 s under 100 mW cm^−2^. AgNW films were fabricated on BHC substrates using an automatic coating machine equipped with an OSP-25 rod (BEVS 1811/2). Specifically, the coating rate and area were set at 120 mm s^−1^ and 12 × 18 cm^2^, respectively. BHC/AgNW TCFs were obtained by drying at 80 °C for 5 min. After that, an FSA (coating thickness of 5 µm) was utilized to coat acrylic resin (1.2 wt%) on the BHC/AgNW TCF substrate. The BHC/AgNW/THC TCF was obtained after drying at 80 °C for 1 min and UV irradiating for 10 s under 100 mW cm^−2^.

### 2.4. Characterization

A scanning electron microscope (SEM) (Sirion 200 FEG, FEI Co., Ltd., Hillsborough, OR, USA) was used to characterize the morphology of AgNWs and TCFs. A Shimadzu Solid Spec 3600 UV-vis-NIR spectrometer (Shimadzu Co., Ltd., Kyoto, Japan) was used to determine the optical reflectance and transmittance spectra with an integral sphere detector. A four-point probe (RTS-9, Guangzhou Four Probe Technology Co., Ltd., China) was used to measure the sheet resistance values. Atomic force microscopy (AFM) was used with an MM8-SYS Bruker AXR scanning probe microscope (Bruker Daltonics Co., Ltd., Madison, WI, USA) to characterize the morphology and roughness of thin films. The aging test was conducted by exposing AgNW TCFs to lab atmosphere. The results were recorded every 3 days for 3 months. A QHQ-A pencil hardness testing device from China was used to measure the pencil hardness of films, in which the sample was tested with a 500 g load. A 3 mm line in five separate areas was used to test the hardness. The hardness of the sample was identified as the pencil hardness that left scratches on fewer than two lines. A nanoindenter (G200, Agilent Technology Co., Ltd., Santa Clara, CA, USA) equipped with a continuous stiffness measurement module was used to analyze the hardness and elastic modulus of the film. For all indentations, a constant strain rate (0.05 s^−1^) loading with diamond indenter (Berkovich, Agilent Technology Co., Ltd., Santa Clara, CA, USA) was used. The indentation depth used for a specimen was always in a range of 0–2 µm, and thus below 20% of the film thickness. It was tested 10 times for each sample to get the average value. For mechanical flexibility tests, silver paste was deposited on two sides of TCFs as electrodes. Bending of the TCFs was performed by using a homemade apparatus.

## 3. Results and Discussion

**Figure 1:**
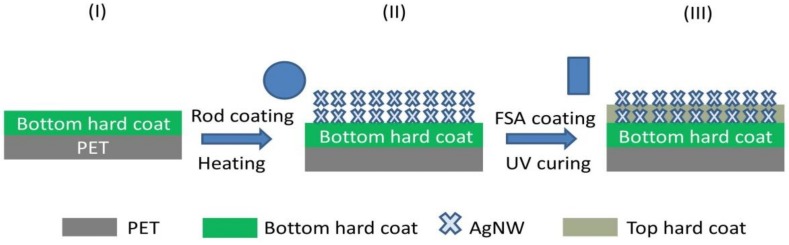
Schematic illustration of fabrication process of bottom hard-coat/silver nanowire/top hard-coat (BHC/AgNW/THC) transparent conductive films (TCFs).

As shown in Figure 1, the BHC/AgNW/THC TCF was prepared by a three-step procedure: (1) preparing a thick BHC layer with 3H hardness onto a PET substrate (PET/BHC), (2) rod-coating of AgNW ink onto the BHC layer (BHC/AgNW), and (3) preparing a thin THC layer as the protective layer onto the BHC/AgNW layer (BHC/AgNW/THC) by a four-sided applicator. For more detailed information see the Materials and Methods section for the formation of BHC/AgNW/THC TCFs.

**Figure 2:**
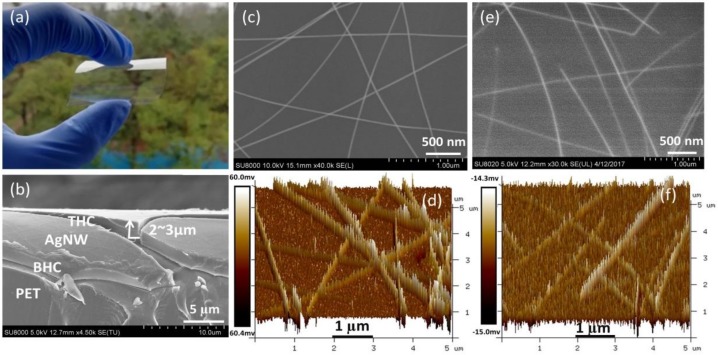
(**a**) Physical photo and (**b**) cross-section SEM image of the fabricated BHC/AgNW/THC TCF. (**c**) Top-view SEM image and (**d**) AFM image of the bare AgNW film coated on the polyethylene terephthalate (PET) substrate. (**e**) Top-view SEM image and (**f**) AFM image of the BHC/AgNW/THC TCF.

Figure 2a shows the physical photo of the fabricated BHC/AgNW/THC TCF, manifesting the high transparency and flexibility. As shown in Figure 2b, the cross-section SEM image clearly exhibits the sandwich structure of BHC/AgNW/THC where the thickness of the BHC layer is about 2–3 µm, and the AgNWs are not completely covered by the THC. Figure 2c,d are images taken using SEM and AFM for bare AgNWs on the PET substrate. The stacking of AgNWs creates a rough surface on the TCF that can easily lead to short circuit when used in electronic devices. The bare AgNW film had a root mean square (RMS) roughness of 7.26 nm and its highest peak–valley range (R_pv_) was 76 nm. In contrast, AgNWs embedded between two HC were smooth with an RMS roughness of 1.98 nm and an R_pv_ of 22 nm, which is beneficial for optoelectronic applications. Based on Figure 2e,f, the morphology of these images indicates that the THC light-activated resin coated by the four-sided applicator permeates deep into the AgNW network and fills in its holes, as well as those at the interface of the AgNWs and the BHC substrate.

**Figure 3:**
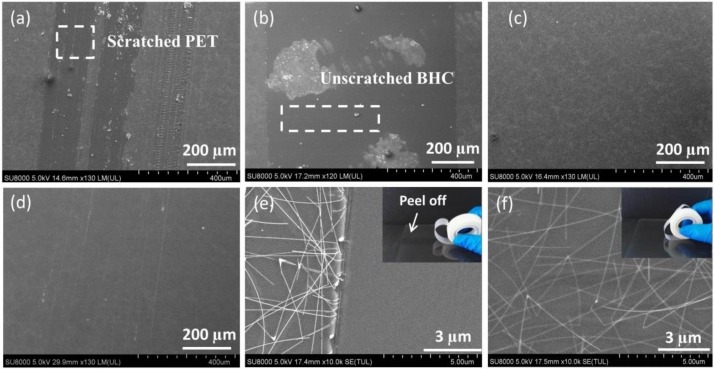
SEM images of (**a**) the bare AgNW TCF, (**b**) the BHC/AgNW TCF and (**c**) the BHC/AgNW/THC TCF after scratching with a 3H pencil. (**d**) SEM image of the BHC/AgNW/THC TCF after scratching with a 4H pencil. SEM images of (**e**) the bare AgNW TCF after the 3M taping test once, and (**f**) BHC/AgNW/THC TCF after the 3M taping test 50 times.

Hardness of AgNW TCFs is a key factor for practical applications. The hardness reflects the ability of the TCF to resist external hard pressure, which has significant impact on the scratch resistance of TCF. However, the research on hardness of AgNW TCFs is still limited. Here, a pencil test was used to investigate the hardness performance of bare AgNW TCFs and BHC/AgNW/THC TCFs. Figure 3a shows the low magnification SEM image of bare AgNW TCF after 3H pencil scratching. The pencil scratching partially peeled off the bare AgNW TCF, and the PET substrate was scratched. It suggests a weak adhesion of bare AgNWs to PET, and the PET layers underneath with lower hardness than 3H. To increase the hardness of the TCF, the BHC/AgNW TCF was prepared. Figure 3b shows the low magnification SEM image of the BHC/AgNW TCF after 3H pencil scratching. The AgNW TCF was peeled off but the BHC substrate was unscratched, indicating that the substrate can resist the 3H pencil load, but the overall hardness and scratch resistance of the BHC/AgNW TCF needs to be further optimized due to the weak binding force between the AgNWs and the BHC substrate. Figure 3c shows that the BHC/AgNW/THC TCF remained intact after the 3H hardness test, indicating that the THC increased adhesion between the AgNWs and the 3H BHC, and prevented the relative sliding of the AgNWs from the BHC, which is beneficial for the overall hardness of the BHC/AgNW/THC TCF. The adhesion force of BHC/AgNW/THC TCF was evaluated by a 3M tape test, as shown in Figure 3f. The AgNW layer of the BHC/AgNW/THC TCF did not detach from the substrate after the 50-times-of-taping test, exhibiting a relatively strong adhesion of AgNW networks to the substrate; while the uncovered AgNWs on the substrate were peeled off completely after testing once (Figure 3e). Figure S2 shows that the resistance of the BHC/AgNW/THC TCF remained relatively stable during the 50-times-of-taping test. This is because the THC layer formed a tight cap over the AgNWs, which was inserted inside the BHC layer to ensure that the layers remained connected. The 4H pencil scratching was also used to test better hardness performance, but the results show that scratches appeared on the substrate (Figure 3d). Thus, the BHC/AgNW/THC TCF can resist 3H pencil scratches and has a 3H hardness level. The increased hardness of the BHC/AgNW/THC TCF can be explained by the fact that the overlying HC protects the AgNW layer due to a high durability and low shear force that results in slipping. Moreover, due to the spring effect, the 3H bottom HC under the layer of AgNWs spread out the contact force and shear force on the overlying layer.

**Figure 4:**
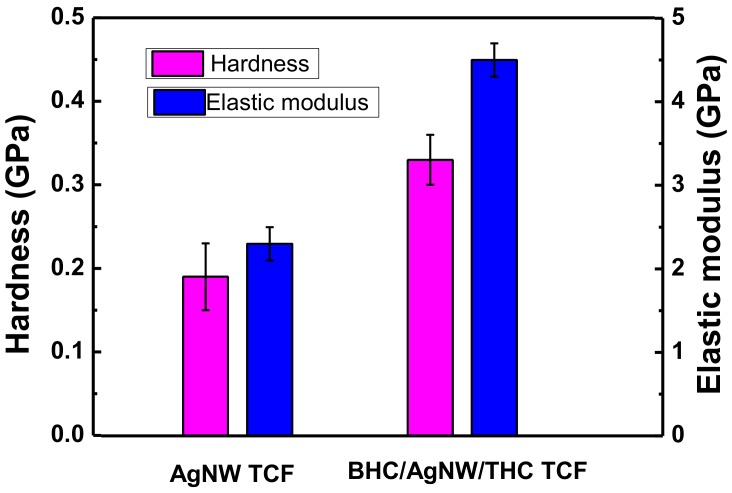
Hardness and elastic modulus values of AgNW TCF and BHC/AgNW/THC TCF measured by nanoindentation.

In addition to the adhesion measurements, the hardness and elastic modulus were also investigated using nanoindenter measurements to provide quantitative information. The BHC/AgNW/THC TCF showed greater hardness and elastic modulus than the bare AgNW TCF indicating better scratch-resistance (Figure 4). This improvement demonstrates that the hardening treatment greatly influenced the properties of TCF.

The optical and electrical performances of the BHC/AgNW/THC and bare AgNW TCFs were estimated (Figure S1). Compared with AgNW TCF, the hardening-treated BHC/AgNW/THC TCF exhibited negligible change of light transmittance but 28% lower haze, owing to the HC layer that reduced light scattering. Though the sheet resistance increases slightly, it does not affect the application in optoelectronic devices (such as touch panels). Aspects such as sheet resistance, transmittance and haze at 550 nm, and pencil hardness of AgNW-based hybrid films are compared in Table 1.

To further clarify the improvement mechanism by the HC, additional coatings were applied on the surface of PET to improve the hardness and abrasion resistance of PET. Various types of FSA with different coating thicknesses (10 µm, 15 µm and 20 µm) were utilized to coat acrylic resin (25 wt% RSH-P2015) on PET substrates. As shown in Figure S3a, the warpage at the interface occurred to different degrees, depending on the coating thickness. In this experiment, PET/BHC with 15 µm FSA coating thickness was applied and the residual stress was ignorable. As shown in Figure S3b,c, the film structure of AgNWs at the shrinkage interface where the residual stress of BHC/AgNW/THC TCF was concentrated was not influenced. This idea is supported by better optoelectrical performance of BHC/AgNW/THC TCF compared to other AgNW based hybrid films (Table 1) as well as improved durability and flexibility as discussed in the following section.

**Figure 5:**
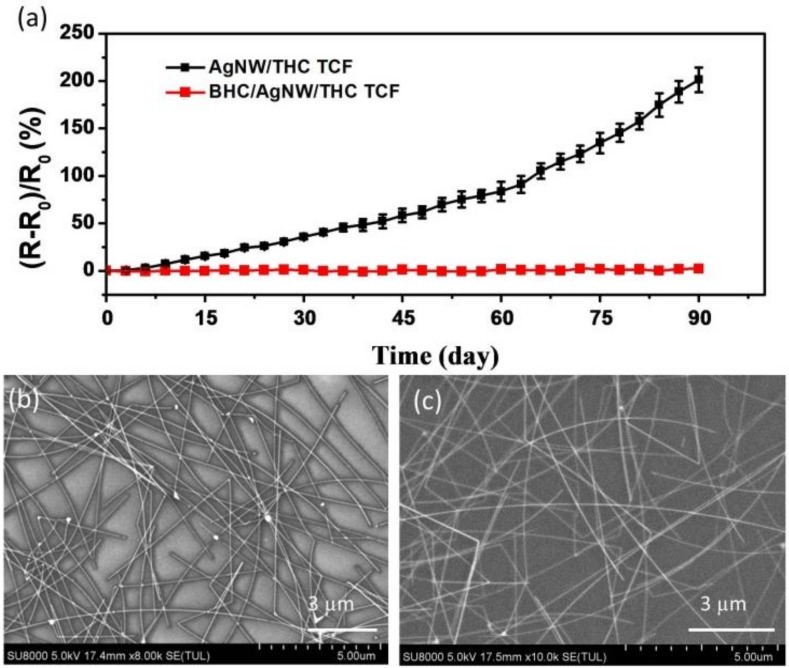
(**a**) Relative resistance changes of the bare AgNW TCF and the BHC/AgNW/THC TCF, observed during 90 days of long-term stability tests. The relative change of resistance is expressed as (R-R_0_)/R_0_, where R is the resistance after the stability test and R_0_ is the resistance before the stability test. SEM images of the (**b**) bare AgNW TCF and (**c**) BHC/AgNW/THC TCF after the long-term stability test.

Durability assessments regarding the aging time of the HC protective coating layer were completed through measuring the variation of resistance. The AgNW TCF and BHC/AgNW/THC TCF were placed at lab conditions in a specific temperature (15–30 °C) and humidity (25–30%) range for 90 days. Figure 5a shows that the resistance of the AgNW TCF increased by above 200% after exposure in air for 90 days. Air exposure easily oxidizes AgNWs [18,24]. As shown in Figure 5b, silver oxides formed on the surface of the AgNWs and resulted in a higher resistance. Conversely, the resistance of the BHC/AgNW/THC TCF in which the HC protected the AgNWs was almost unchanged, and there were no silver oxides on the BHC/AgNW/THC TCF. Clearly, in comparison to the bare layer of AgNWs, the coatings with hard protective layers demonstrated superior durability throughout aging.

Flexibility is critical for successful device applications; therefore, the impact of hardening treatment on the flexibility of AgNW TCFs must be evaluated. The mechanical flexibility of a bare AgNW TCF and the BHC/AgNW/THC TCF is compared in Figure S4. As shown in Figure S4a, with the decrease of bending radius (see inset picture), the resistance growth rate of the BHC/AgNW/THC TCF slowed down gradually, even though it was larger at the beginning stage. In contrast, the resistance growth rate of bare AgNW TCF was constantly increasing, which may be due to the loose AgNW network sliding over the PET substrate. The BHC/AgNW/THC TCF clearly showed a better mechanical flexibility under extreme bending at a 0.5 mm radius. This is due to the strongly bonded HC matrix and AgNW networks, which could prevent the occurrence of sliding at the interface. Repeated bending test of the bare AgNW TCF and BHC/AgNW/THC TCF is shown in Figure S4b, and the value for resistance change of the bare AgNW TCFs after 5000 bending cycles with a bending radius of 1.0 cm was 6.3%. However, the value of hardening-treated BHC/AgNW/THC TCF increased by 12.4% under the same condition, suggesting that the hardening treatment affected the repeated mechanical flexibility of the AgNW films. It should be pointed out that the repeated mechanical flexibility performance of BHC/AgNW/THC TCF was still better than that of AZO/AgNW/AZO TCF with a sandwich structure [17]. UV curing acrylic resin coatings on the surface of thin films are commonly used to enhance the hardness and abrasion resistance of thin films, but the introduction of hardening coatings usually affects the flexibility of thin films [25]. Therefore, how to optimize the composition of UV ink and balance the relationship between film hardness and flexibility needs further research.

Overall, the hardening treatment not only improved the optical haze, scratch resistance, and adhesion stability of AgNW TCFs, but also led to a passable mechanical flexibility.

## 4. Conclusions

In summary, a facile approach has been developed to prepare a sandwich-structured BHC/AgNW/THC TCF by embedding a AgNW layer between two HC layers. The BHC/AgNW/THC TCF shows high scratch resistance with the hardness of 3H, and enhanced adhesion to the substrate. In addition, the BHC/AgNW/THC TCF exhibits low haze (1% at 550 nm) and negligible change of light transmittance under low sheet resistance (72 ± 4.0 Ω/sq). Furthermore, as a result of the superior gas barrier properties of the HC layers, the BHC/AgNW/THC TCF has excellent long-term stability. Therefore, using the hard coating layers can enhance the performance of BHC/AgNW/THC TCF. This research proposes a realistic method to address difficulties regarding real-world applications with the need of high definition, functional strength, and physical robustness for optoelectronic devices.

## Figures and Tables

**Table 1:** Comparison with previous studies of AgNW-based hybrid films.

TCF	Rs (Ω/sq)	T%	Haze	Pencil Hardness
GR/AgNW/GR [15]	19.9 ± 1.2	88.6%	None	None
AgNW/CNT/rGO [16]	45–75	78%–94%	None	None
AZO/AgNW/AZO [17]	27.6	80.5%	14.9%	None
AgNWs/PEDOT:PSS [18]	75	>90%	1.21%	None
BHC/AgNW/THC (This work)	72	90.6%	1%	3H

## Supplementary Materials

The following are available online at https://www.mdpi.com/2079-4991/9/4/557/s1. Figure S1. UV-Vis curves to characterize the transmittance and haze of TCFs fabricated by the (a) bare AgNW layer and (b) BHC/AgNW/THC layer, respectively. The sheet resistance statistic distribution of the (c) bare AgNW TCF and (d) BHC/AgNW/THC TCF, respectively. Figure S2. Relative changes in the resistance after the 3M taping test, once for the bare AgNW TCF and 50 times for the BHC/AgNW/THC TCF. Figure S3. (a) The picture of PET/BHC substrates with 10 µm, 15 µm and 20 µm coating thickness. (b) Normal and (c) enlarged interface SEM image manifesting the intact AgNW film structure. Figure S4. (a) Relative resistance changes of the bare AgNW TCF and the BHC/AgNW/THC TCF as a function of bending radius. (b) Relative resistance changes of the films under bending tests with 5000 bending cycles under bending radius of 1 cm.
