# Supplementary material for: Sandwich-Structured Silver Nanowire Transparent Conductive Films with 3H Hardness and Robust Flexibility for Potential Applications in Curved Touch Screens
Source: Nanomaterials (Basel). 2019 Apr 5;9(4):557. doi: 10.3390/nano9040557 (PMC6523603; doi:10.3390/nano9040557)
Supplement: Supplementary file 1 [file nanomaterials-09-00557-s001.pdf]

Supporting Information

Sandwich-Structured Silver Nanowire Transparent Conductive Films with 3H Hardness and Robust Flexibility for Potential Applications in Curved Touch Screens

Xikun Chu [1,2], Jingqi Tao [1,2], Shuxin Li [1], Shulin Ji [1,*] and Changhui Ye [1,3,*]

1 Key Laboratory of Materials Physics, Anhui Key Laboratory of Nanomaterials and Nanotechnology, Institute of Solid State Physics, Chinese Academy of Sciences, Hefei 230031, China; xikunchu@163.com (X.C.); jqtao127@163.com (J.T.); lishuxin@issp.ac.cn (S.L.)

2 Science Island Branch of Graduate School, University of Science and Technology of China, Hefei 230026, China

3 College of Materials Science and Engineering, Zhejiang University of Technology, Hangzhou 310014, China

* Correspondence: slji@issp.ac.cn (S.J.), chye@zjut.edu.cn (C.Y.); Tel.: +86-551-65591923 (S.J.)

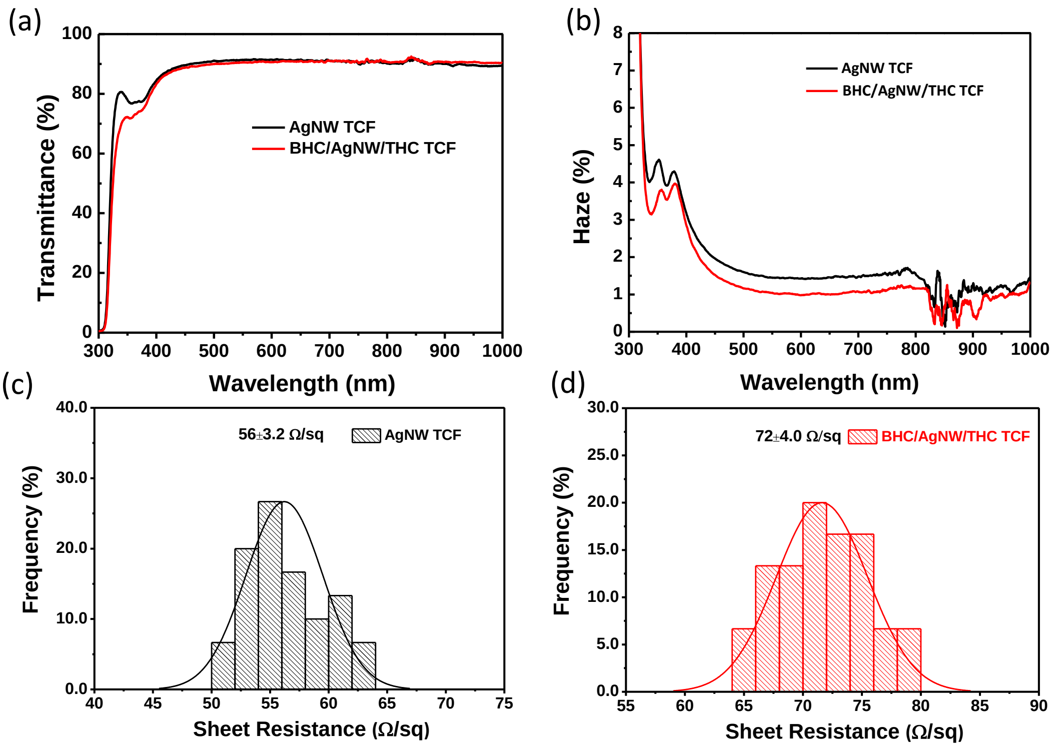

Figure S1. UV-Vis curves to characterize the transmittance and haze of TCFs fabricated by the (**a**) bare AgNW layer and (**b**) BHC/AgNW/THC layer, respectively. The sheet resistance statistic distribution of the (**c**) bare AgNW TCF and (**d**) BHC/AgNW/THC TCF, respectively. The optical transmittance, haze and sheet resistance of the bare AgNW TCF were 91.6%, 1.4% at 550 nm and 56 ± 3.2 Ω/sq; while after hardening process, the values for BHC/AgNW/THC TCF were 90.6%, 1% at 550 nm and 72 ± 4.0 Ω/sq, respectively.

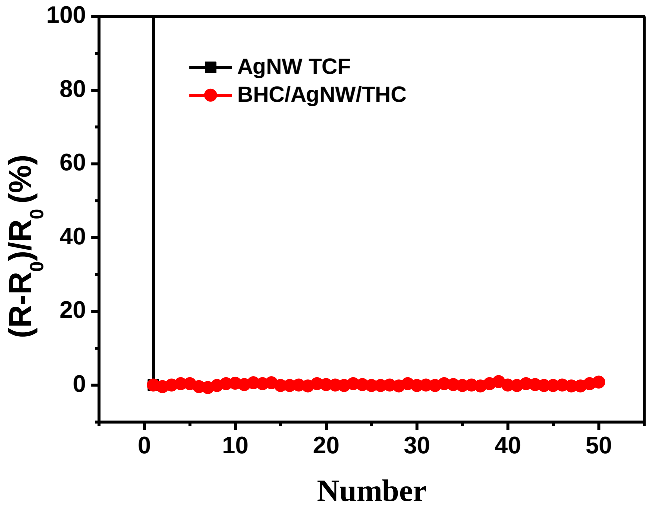

Figure S2. Relative changes in the resistance after the 3M taping test, once for the bare AgNW TCF and 50 times for the BHC/AgNW/THC TCF.

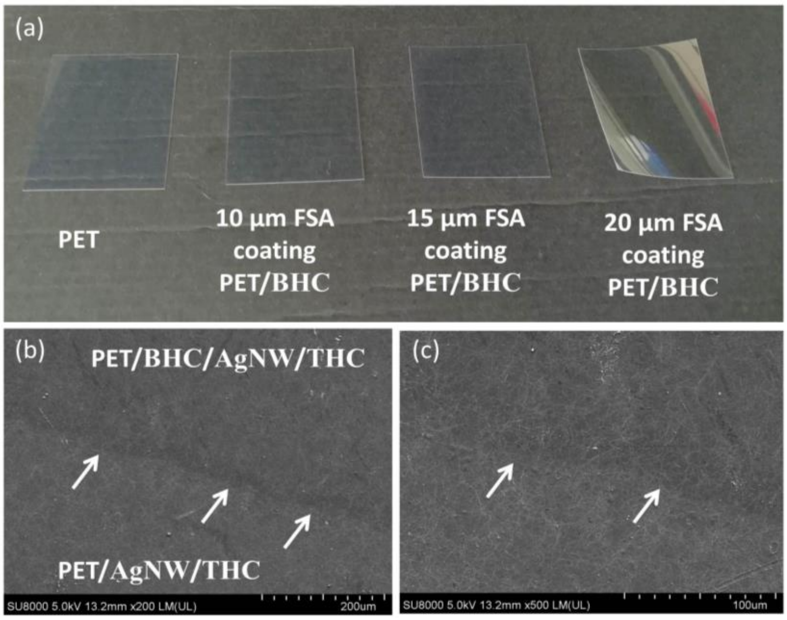

Figure S3. (**a**) The picture of PET/BHC substrates with 10 μm, 15 μm and 20 μm coating thickness. (**b**) Normal and (**c**) enlarged interface SEM image manifesting the intact AgNW film structure.

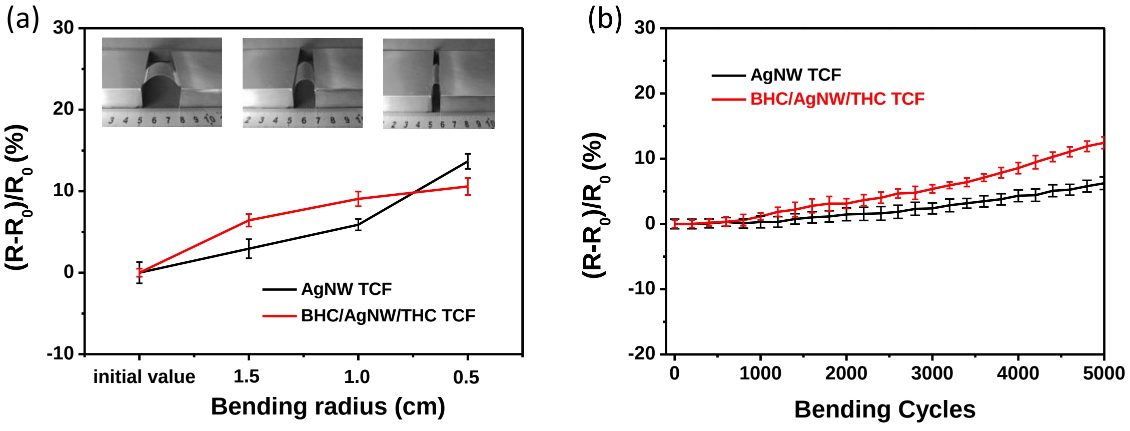

Figure S4. (**a**) Relative resistance changes of the bare AgNW TCF and the BHC/AgNW/THC TCF as a function of bending radius. (**b**) Relative resistance changes of the films under bending tests with 5000 bending cycles under bending radius of 1 cm. The relative change in resistance is expressed as $(R-R_0)/R_0$, where R is the resistance after bending tests and R_0 is the resistance before bending.
